# Can genomics shed light on the origin of species?

**DOI:** 10.1371/journal.pbio.3000394

**Published:** 2019-08-30

**Authors:** Chris D. Jiggins

**Affiliations:** Department of Zoology, University of Cambridge, Cambridge, United Kingdom

## Abstract

Evolutionary biologists are increasingly using population genetic variation across genomes to address questions around the origin and ongoing evolution of species. Patterns of differentiation between closely related species are highly variable across the genome, and a wide variety of processes contribute to that variation. There is an emerging pattern of parallelism, whereby different species pairs in groups of related species show similar differentiation patterns across their genomes, offering an opportunity to test hypotheses regarding the processes underlying species differentiation. A recent study used both simulations and empirical data to investigate different forms of selection in a radiation of monkeyflowers. The parallel patterns emerged very rapidly after divergence and could not be readily explained by selection for removal of deleterious mutations but instead likely results from some combination of adaptive evolution, species incompatibilities, and ongoing gene flow. Overall, an emerging pattern is that there may be a surprising degree of predictability in the genetic architecture of species differences across groups of related species.

## Abstract 2

Genetic variation across genomes can be used to address long-standing questions over the origin and ongoing evolution of species. This Primer explores the implications of one such study that is suggesting a surprising repeatability in patterns of genetic divergence across genomes and between species.

## Introduction

A major goal of speciation genomics is to understand the evolutionary processes responsible for patterns of genetic variation between populations and species as they diverge. As it becomes increasingly feasible to assemble reference genomes for new species and generate sequence data for large numbers of individuals from natural populations, this has become a rapidly burgeoning area of evolutionary biology research [1,2]. Broadly, the goals are to understand the processes and genetic architecture underlying the formation of new species, and ultimately, of biodiversity more widely. As we trace any lineage back through time, it has been through many speciation events—indeed, because speciation is a gradual process, it could be argued that virtually all populations are in the process of speciation with a close or distant relative at any moment in time. Patterns emerging from speciation genomic studies are therefore important for understanding speciation but also for genome evolution more generally.

Questions that can be answered by speciation genomics broadly fall into two classes. On the one hand, many researchers are interested in identifying the specific loci that control species differences or cause reproductive isolation. These are sometimes termed the ‘speciation genes’ that are responsible for the evolution of a new species. Outstanding questions include whether particular classes of genes play an important role and what kinds of mutations are more important (e.g., regulatory or coding sequence). We can also explore whether the novel variation for evolution is coming from de novo mutations or from existing variants, either through hybridisation or standing variation. On the other hand, there are a class of questions that can be answered without the need to identify specific loci under selection. These include how many such loci are needed for the evolution of new species and how they are distributed through the genome. In addition, occasional hybridisation between species can lead to exchange of genes, and genomic studies can identify and quantify such gene flow between populations. The timing of such gene flow can potentially distinguish between alternative geographic scenarios for speciation, such as identifying periods of complete geographic isolation, and shed light on the degree to which genomes evolve independently of one another once speciation has begun. In a similar vein, patterns of divergence can shed light on the strength of selection against mixing and therefore the degree to which genomes have evolved incompatibilities.

The theoretical framework underlying speciation genomics has itself evolved rapidly over the past few years. The early focus was on understanding variation in patterns of differentiation across the genome, often measured using a statistic known as *Fst* [3]. It was hypothesised that such variation would broadly represent the interaction of two forces: on the one hand, divergent selection pushing genomes apart (leading to high *Fst*), and on the other, mixing of genomes due to hybridisation pulling them back together (low *Fst*). Thus, it was envisaged that by sequencing populations of closely related species living together, we would be able to readily identify those genomic regions of high differentiation that are responsible for speciation. *Fst* is a useful and easily interpreted statistic, and where strong selection acts against a background of high gene flow, *Fst* outliers are indeed the easiest approach for identifying loci under selection [4]. Sadly however, where gene flow is reduced by reproductive isolation during speciation, things turned out to be rather more complicated.

In fact, there are many processes that influence variation in differentiation between populations across the genome, only some of which are directly relevant to understanding speciation [5–7]. One problem is that *Fst* is a measure of the relative diversity between versus within populations and as such can be influenced by evolutionary processes within as much as between populations. A peak in *Fst* in a genomic region might be due to divergent selection maintaining species differences, but equally it could be due to selection reducing overall variability within one or both of the populations. This reassessment led to a general scepticism over the interpretation of *Fst* peaks as being regions involved in speciation. One study system that epitomised this changing view were pied and collared flycatchers, closely related bird species that are known to hybridise occasionally in central Europe. Peaks of divergence between these species were initially interpreted as regions responsible for speciation but later as being explicable at least in part by ongoing within species selection [8,9].

One approach to gain better insight is to expand out from the pairwise comparisons used to calculate *Fst*. Variation in phylogenetic relationships across the genome among multiple lineages can instead be used to identify regions supporting different hypotheses. For example, the ABBA-BABA tests use a four-taxon phylogeny to count single nucleotide patterns consistent with gene flow between species [10,11]. In other cases, the full phylogeny is reconstructed in windows across the genome. For example, in the recent study of monkeyflower species, some regions of the genome consistently showed a better agreement with the expected species tree than others [12]. Potentially, those regions that follow most closely relationships predicted by the ecology and morphology of the species might be those that control species-specific traits. A similar analysis in *Heliconius* butterflies found that some chromosomes showed clustering by expected species relationships but others were more consistent with recent gene flow, clustering populations instead by geographical proximity rather than by species [13]. Including multiple lineages in the analysis, as opposed to just looking at pairwise comparisons of species, can therefore shed additional light on the processes involved.

An emerging pattern is that variation across the genome often shows strong parallelism. In other words, when independent species pairs are compared across a group of species, there is often a similar pattern of variation across the genome. Such parallelism has been observed in *Heliconius* butterflies, flycatchers, stickleback fish, sunflowers, and Darwin’s Finches but is especially prominent in the monkeyflowers [1,9,12,14–16]. One fundamental reason for such repeated patterns is that genetic variation is correlated with genomic features, such that regions of high gene density and low recombination often show a pattern more concordant with the species relationships [17]. High gene density implies more sites that are likely to be subject to selection, and low recombination means that such selection has a greater influence on surrounding genomic regions through linkage. The challenge however is to disentangle the different forms of selection that could lead to such patterns.

There are a number of possible explanations for parallelism, and the monkeyflower study attempts to disentangle them. The first scenario invokes a process known as ‘background selection’, which is the ongoing removal of harmful mutations by natural selection and its influence on linked sites [5,18]. This will have a stronger effect in regions of high gene density (where mutations are more likely to be functional) and in regions of low recombination, where such selection is more strongly linked to surrounding genetic variation. Background selection should lead to reduced genetic variation and hence increased *Fst* in low recombination regions of the genome, as is commonly observed. However, it is unclear whether this is a sufficiently powerful force to generate the patterns seen over short timescales.

The second scenario is repeated positive selection in diverging lineages. When a beneficial mutation is selected, it removes genetic variation from surrounding regions in a process known as a selective sweep. It has been considered improbable that positive selection would be repeated in the same genomic regions across many lineages [18], because the rate of beneficial mutation is too low to produce such repeated patterns. However, if existing variants are selected for in parallel across multiple populations, without the need for new mutations, this could lead to similar patterns of selection across multiple species pairs [19]. This is seen, for example, in stickleback fish that repeatedly adapt to freshwater habitats using the same ancient alleles [14]. Similarly, the same genes are likely to be repeatedly targeted in adaptation, which could lead to parallelism even if the precise mutations are different in each population [20]. Repeated positive selection could therefore also lead to parallel patterns of reduced genetic variation in the same genome regions. Such positive selection may be directly involved in the divergence of species but could also represent other forms of adaptation.

Third, the speciation process itself could be repeatable across lineages. If there is a common genetic architecture underlying reproductive isolation, with the same genes repeatedly causing incompatibilities between genomes, this might lead to correlated patterns. This would be especially the case if there is ongoing gene flow between species that mixes genetic variation at regions not showing such incompatibilities. A common genetic architecture of speciation seems likely among closely related species, in which the same incompatibility alleles might influence multiple species pairs. Even if incompatibility loci were not actually the same loci, or were randomly distributed across the genome, variation in recombination rate could lead to predictable patterns, because high recombination will separate selected loci from neutral variation more rapidly, allowing higher rates of gene flow between species [13,17,21].

Finally, patterns of genetic variation may predate the common origin of multiple species, such that apparently independent lineages in a species group may not be truly independent of one another—parallelism could reflect a shared evolutionary history rather than deterministic ongoing processes. A long history of background and positive selection can lead to very heterogeneous pattern of genetic diversity across the genome even before two species split, and this shared legacy of past selection can lead to parallel patterns that are nothing to do with speciation itself [18].

Stankowski and colleagues address these different hypotheses using a combination of empirical data and simulations. By simulating some regions that were entirely neutral and others containing mutations under different forms of selection, the authors were able to compare the likelihood of different scenarios. Broadly, the conclusion of these simulations was that background selection was too weak to generate correlated patterns on the timescale of the monkeyflower radiation [12]. Instead it seems likely that some combination of positive selection, species incompatibilities, and ongoing gene flow are needed to explain the similarity of patterns seen across the radiation. Nonetheless, simulations over longer time scales might be necessary to fully describe the complexity of shared evolutionary history prior to, as well as during, speciation.

Overall, it is clear that the processes influencing divergence between species are far more complex than was initially envisaged. However, the current study demonstrates how the field is maturing and developing an appreciation of this complexity. Simulations are likely to provide a powerful means of testing between alternative hypotheses when the scenarios are complex and involve multiple species. To complement this approach, we will also need to develop methods by which we can simultaneously compare alternative scenarios in windows across the genome, and model-fitting approaches are now being developed to do just that [22]. These are computationally intensive and unlikely to be applicable across many species simultaneously but complement the more descriptive approaches highlighted above. In summary, this rapidly growing field of evolutionary biology is suggesting that the genetic basis for speciation may be far more predictable than has been previously envisaged, a pattern similar to that for the genetic basis for adaptation more generally [20]. However, in speciation, it is not yet clear to what extent this predictability might be due to selection on the same specific genes during speciation or alternatively due to selection acting in a shared genomic environment of recombination rate and gene density. There is still plenty of scope for future work to tease apart these different processes.
